# Association between diabetes mellitus and bone mineral density in adults in Yanbu, Saudi Arabia: a retrospective cross-sectional study

**DOI:** 10.25122/jml-2026-0119

**Published:** 2026-09

**Authors:** Layal Khalid Jambi, Abdulaziz Lafi Alluqmani, Ahmed Ali Mahzari, Khulud Aedh Al-Ghamdi, Ahmed Abdullah Alharbi, Osama Hamad Jaafari, Emadeldin Abdeljabar Bashir, Rafal Khalid Jambi

**Affiliations:** 1 Radiological Sciences Department, College of Applied Medical Sciences, King Saud University, Riyadh, Saudi Arabia; 2 Medical Imaging Department, Royal Commission Hospital, Yanbu, Saudi Arabia; 3 Health Informatics Department, College of Health Sciences, Prince Musaid bin Abdulrahman University, Dammam, Saudi Arabia

**Keywords:** bone mineral density, diabetes mellitus, DXA, osteoporosis, osteopenia, lumbar spine, femoral neck, Saudi Arabia, Yanbu, AGEs, advanced glycation end-products, BMD, bone mineral density, BMI, body mass index, DM, diabetes mellitus, DXA, dual-energy X-ray absorptiometry, FN, left femoral neck, FRAX, Fracture Risk Assessment Tool, LS, lumbar spine, MAPK, mitogen-activated protein kinase, NF-κB, nuclear factor kappa B, RAGE, receptor for advanced glycation end-products, SD, standard deviation, T1DM, type 1 diabetes mellitus, T2DM, type 2 diabetes mellitus, TBS, trabecular bone score, WHO, World Health Organization

## Abstract

Diabetes mellitus and osteoporosis are prevalent chronic conditions with a substantial global public health burden. Their relationship remains incompletely understood, particularly in Saudi Arabia. This retrospective cross-sectional study examined the association between diabetes mellitus and bone mineral density (BMD) at the lumbar spine and left femoral neck among adults attending the dual-energy X-ray absorptiometry (DXA) service at Royal Commission Hospital, Yanbu, Saudi Arabia. Overall, 107 adults undergoing DXA scanning between 2018 and 2025 were classified as diabetic (*n* = 92) or non-diabetic (*n* = 15). BMD, T-scores, and Z-scores were recorded for the lumbar spine (L1–L4) and femoral neck, and bone status was classified according to World Health Organization T-score criteria. No statistically significant differences in lumbar spine or femoral neck BMD were observed between groups (all *P* > 0.05). Among patients with diabetes, lumbar spine osteoporosis and osteopenia were present in 39.1% and 40.2%, respectively, while femoral neck osteopenia and osteoporosis were identified in 51.1% and 8.7%. Overall, 40.2% met osteoporosis criteria at one or both sites. Within the diabetic group, body mass index (BMI) correlated positively with lumbar spine T-score (*r* = 0.445; *P* < 0.001) and femoral neck T-score (*r* = 0.376; *P* < 0.001), whereas age correlated negatively with femoral neck T-score (*r* = −0.208; *P* = 0.046) and BMD (*r* = −0.213; *P* = 0.042). Reduced BMD was common among patients with diabetes, with higher BMI associated with better bone status and advancing age associated with lower femoral neck BMD. The absence of between-group differences should be interpreted cautiously given the small non-diabetic comparison group.

## INTRODUCTION

Diabetes mellitus (DM) is a major global public health challenge characterized by chronic hyperglycemia resulting from defects in insulin secretion, insulin action, or both. The International Diabetes Federation estimated that approximately 537 million adults worldwide were living with diabetes in 2021, a figure projected to rise to 783 million by 2045 [1]. Within Saudi Arabia, the burden is particularly pronounced: national surveys indicate a diabetes prevalence exceeding 17% among adults, with rates continuing to rise in conjunction with urbanization, dietary transitions, and escalating obesity [2]. The western coastal region, including Yanbu, mirrors this national trajectory, making diabetes-related comorbidities a pressing local concern.

Osteoporosis, characterized by reduced bone mass and microarchitectural deterioration of bone tissue, affects an estimated 200 million individuals globally and is increasingly recognized as a serious comorbidity in individuals with DM [3]. The World Health Organization (WHO) defines osteoporosis as a T-score of −2.5 standard deviations (SD) or more below the young adult mean, as measured by dual-energy X-ray absorptiometry (DXA); it predisposes patients to fragility fractures, chronic pain, functional disability, and increased mortality [4]. Saudi Arabia has witnessed a rising prevalence of osteoporosis, with hospital-based surveys reporting rates of 52–64% among patients undergoing DXA scanning [5,6].

The association between DM and bone health is complex and not fully elucidated. Type 1 diabetes mellitus (T1DM) has been consistently linked to reduced bone mineral density (BMD) because insulin deficiency impairs osteoblast function [7,8], and experimental models confirm that insulin-deficient states induce progressive trabecular bone loss and reduced whole-bone mechanical strength [9]. By contrast, type 2 diabetes mellitus (T2DM) presents a paradox: BMD is frequently reported as normal or even elevated compared with non-diabetic controls, yet fracture risk is markedly increased [10]. This discordance between BMD and fracture risk in T2DM is partly attributed to qualitative deterioration of bone material properties—mediated principally by the accumulation of advanced glycation end-products (AGEs) within the bone matrix—rather than loss of mass per se [11,12].

Several pathophysiological mechanisms have been proposed to explain impaired skeletal health in diabetes. Chronic hyperglycemia promotes the non-enzymatic glycation of type I collagen, generating pentosidine and other AGE cross-links that stiffen collagen fibrils, impair mineralization, and reduce bone ductility [12]. AGEs interact with the receptor for AGEs (RAGE), activating nuclear factor kappa B (NF-κB) and mitogen-activated protein kinase (MAPK) signaling cascades, which amplify inflammatory cytokine production, promote osteoblast apoptosis, and stimulate osteoclastogenesis [13,14]. Furthermore, hyperglycemia-induced oxidative stress, microvascular disease, and peripheral neuropathy collectively contribute to increased cortical porosity and elevated fall risk, further elevating fracture probability [12,13]. Higher body mass index (BMI), which is disproportionately prevalent among individuals with T2DM, may attenuate bone loss through mechanical loading and adipose tissue endocrine activity, partially explaining why some studies report preserved or elevated BMD in this population [15–17].

Data from the Royal Commission Hospital, Yanbu, Saudi Arabia, on the diabetes–BMD relationship are sparse. A study from Riyadh reported osteoporosis in 36.8% of Saudi adults with diabetes and identified higher BMI as a protective factor [18], while a Jeddah study of female patients with diabetes found rates of osteoporosis reaching 29.4% and osteopenia up to 40%, with advancing age and vitamin D deficiency as key risk factors [19]. Yanbu, an industrial coastal city in the Madinah region, lacks dedicated epidemiological data on this relationship, creating a gap in regional knowledge relevant to clinical care planning.

The present study was therefore designed to investigate the association between diabetes mellitus and BMD, measured at the lumbar spine and left femoral neck by DXA, in adults attending the radiology department of Royal Commission Hospital, Yanbu. Secondary objectives included describing the prevalence of osteoporosis and osteopenia in the diabetic cohort and identifying anthropometric determinants of BMD within this group.

## MATERIAL AND METHODS

### Study design and setting

This was a retrospective cross-sectional study conducted in the radiology department of Royal Commission Hospital, Yanbu, Saudi Arabia. DXA scan records from January 2018 to April 2025 were retrieved and analyzed. Ethical approval was obtained from the Medical Ethics and Research Committee of Royal Commission Hospital, Yanbu (Ref. RCYMC-EA-2023-01), and all patient data were handled in accordance with the Declaration of Helsinki and applicable national privacy regulations. As data were retrospectively extracted from existing clinical records and fully anonymized before analysis, the requirement for individual informed consent was waived.

### Participants

Records from adult patients (age ≥ 15 years) who underwent DXA scanning for bone density assessment during the study period were eligible for inclusion. Patients were classified as diabetic or non-diabetic based on the clinical designation recorded in their medical record at the time of the scan. Records with incomplete DXA data (missing BMD values, T-scores, or Z-scores at either site) or missing demographic information were excluded. After applying these criteria, 107 patient records were included: 92 in the diabetic group and 15 in the non-diabetic group.

### Data collection

The following variables were extracted from each patient record: year of DXA scan, age (years), sex, height (meters), weight (kilograms), BMI (kg/m^2^), diabetic status, nationality, lumbar spine BMD (g/cm^2^), lumbar spine T-score and Z-score (reflecting the L1–L4 region), left femoral neck BMD (g/cm^2^), and left femoral neck T-score and Z-score. BMI values were cross-checked against height and weight data to identify implausible entries; a small number of height entries recorded in centimeters were converted to meters. Several BMD values in the dataset were recorded as three-digit integers (e.g., 821) presumed to represent values with an omitted decimal point (e.g., 0.821 g/cm^2^); these were corrected to the decimal form before analysis.

### Bone mineral density assessment

BMD was measured using DXA (Hologic or equivalent), the internationally accepted gold-standard method for non-invasive BMD assessment and osteoporosis diagnosis [4]. DXA measurements at the lumbar spine and femoral neck have demonstrated high reproducibility, with a precision error of approximately 1–2.5%, and provide quantitative BMD data at skeletal sites most predictive of fracture risk [20]. Bone density status was classified according to WHO T-score criteria as follows: normal (T-score ≥ −1.0), osteopenia (−2.5 < T-score < −1.0), and osteoporosis (T-score ≤ −2.5) [4]. Z-scores, which reflect BMD relative to age- and sex-matched reference populations, were also recorded. For participants younger than 50 years, Z-scores were considered the primary densitometric reference, as recommended by the International Society for Clinical Densitometry [4].

### Statistical analysis

Statistical analysis was performed using Python (version 3.12) with the pandas, NumPy, and scipy.stats libraries. Continuous variables are presented as mean ± SD or median with interquartile range, where appropriate. Categorical variables are reported as frequencies and percentages. Between-group comparisons of continuous BMD parameters were conducted using independent-samples *t* tests; the Mann–Whitney *U* test was applied as a non-parametric alternative given the small sample size in the non-diabetic group. Categorical BMD classifications were compared between groups using the chi-square test. Pearson product-moment correlation coefficients were computed to examine linear associations between BMI, age, and BMD indices within the diabetic cohort. Statistical significance was defined as *P* < 0.05.

## RESULTS

### Sample characteristics

A total of 107 participants were included: 92 with diabetes (85.9%) and 15 without diabetes (14.1%). The demographic and anthropometric characteristics of both groups are summarised in Table 1. The overall cohort had a mean age of 63.1 ± 11.1 years and was predominantly female (85/107, 79.4%). In the diabetic group, 72 participants (78.3%) were female and 20 (21.7%) were male. The mean age in the diabetic group was 63.6 ± 11.2 years (range 15–85), compared with 60.3 ± 9.5 years (range 45–77) in the non-diabetic group. Mean BMI was 30.5 ± 7.1 kg/m^2^ in the diabetic group and 29.1 ± 5.9 kg/m^2^ in the non-diabetic group. Most participants were Saudi nationals (102/107, 95.3%). No statistically significant differences were observed between the two groups in age, BMI, weight, or sex distribution (all *P* > 0.05).

**Table 1:** Baseline demographic and anthropometric characteristics by diabetes status

Variable	Diabetic group (*n* = 92)	Non-diabetic group (*n* = 15)	*t* / χ^2^	*P*
Age (years)	63.6 ± 11.2	60.3 ± 9.5	1.082	0.281
BMI (kg/m^2^)	30.5 ± 7.1	29.1 ± 5.9	0.723	0.471
Weight (kg)	75.2 ± 18.3	71.3 ± 13.7	0.789	0.432
Female sex, *n* (%)	72 (78.3)	13 (86.7)	0.523	0.470
Saudi nationality, *n* (%)	87 (94.6)	13 (86.7)		

Data are presented as mean ± SD or *n* (%). Continuous variables were compared with the independent-samples *t* test and sex with the chi-square test; nationality was not compared between groups (—). No comparison reached statistical significance (*P* < 0.05). BMI, body mass index; *n*, number of participants; SD, standard deviation.

### Bone mineral density comparisons between groups

Table 2 presents mean BMD values, T-scores, and Z-scores at the lumbar spine (LS) and left femoral neck (FN) for each group. The mean LS BMD was 0.874 ± 0.151 g/cm^2^ in the diabetic group and 0.851 ± 0.153 g/cm^2^ in the non-diabetic group (*t* = 0.544; *P* = 0.588). The mean FN BMD was 0.752 ± 0.140 g/cm^2^ in the diabetic group versus 0.765 ± 0.161 g/cm^2^ in the non-diabetic group (*t* = −0.321; *P* = 0.749). Neither LS nor FN T-scores or Z-scores differed significantly between groups (all *P* > 0.05). Mann–Whitney *U* tests yielded consistent results, confirming the absence of a statistically significant between-group difference in any BMD parameter.

**Table 2:** Bone mineral density parameters at the lumbar spine and left femoral neck by diabetes status

BMD parameter	Diabetic group (*n* = 92)	Non-diabetic group (*n* = 15)	*t*	*P*
LS BMD (g/cm^2^)	0.874 ± 0.151	0.851 ± 0.153	0.544	0.588
LS T-score	−1.95 ± 1.40	−2.07 ± 1.43	0.313	0.755
LS Z-score	−0.59 ± 1.37	−1.00 ± 1.49	1.057	0.293
FN BMD (g/cm^2^)	0.752 ± 0.140	0.765 ± 0.161	−0.321	0.749
FN T-score	−1.10 ± 1.14	−1.05 ± 1.18	−0.150	0.881
FN Z-score	0.06 ± 1.01	−0.08 ± 1.15	0.500	0.618

Data are presented as mean ± SD. Groups were compared with the independent-samples *t*-test. No comparison reached statistical significance (*P* < 0.05). BMD, bone mineral density; FN, left femoral neck; LS, lumbar spine; SD, standard deviation.

### Prevalence of osteoporosis and osteopenia

The distribution of WHO bone density categories for the lumbar spine and left femoral neck is shown in Table 3. Among patients with diabetes, 36 (39.1%) met the T-score threshold for lumbar spine osteoporosis, 37 (40.2%) had osteopenia, and 19 (20.7%) had normal BMD. For the femoral neck, 8 (8.7%) had osteoporosis, 47 (51.1%) had osteopenia, and 37 (40.2%) had normal BMD. Combined across sites, 37 of 92 patients with diabetes (40.2%) had osteoporosis at one or more sites, and 60 (65.2%) had osteopenia at one or more sites. In the non-diabetic group, 8 of 15 participants (53.3%) had lumbar spine osteoporosis and 5 (33.3%) had osteopenia. Chi-square analysis revealed no statistically significant difference in the distribution of bone density categories between diabetic and non-diabetic participants at the lumbar spine (χ^2^ = 1.14; *P* = 0.565) or femoral neck (χ^2^ = 0.244; *P* = 0.885).

**Table 3:** Distribution of WHO bone mineral density categories by diabetes status

BMD category	LS, diabetic (*n* = 92)	LS, non-diabetic (*n* = 15)	LS *P*	FN, diabetic (*n* = 92)	FN, non-diabetic (*n* = 15)	FN *P*
Normal (T ≥ −1.0)	19 (20.7)	2 (13.3)	0.566	37 (40.2)	7 (46.7)	0.885
Osteopenia (−2.5 < T < −1.0)	37 (40.2)	5 (33.3)		47 (51.1)	7 (46.7)	
Osteoporosis (T ≤ −2.5)	36 (39.1)	8 (53.3)		8 (8.7)	1 (6.7)	

Data are presented as *n* (%). Categories follow the WHO T-score criteria. For each site, the distribution across all three categories was compared be-tween groups with the chi-square test; the single *P* value per site is shown in the first row. No comparison reached statistical significance (*P* < 0.05). BMD, bone mineral density; FN, left femoral neck; LS, lumbar spine; T, T-score; WHO, World Health Organization.

### Correlations of BMD with age and BMI in the diabetic group

Pearson correlation analyses within the diabetic cohort (Table 4) revealed that BMI was significantly and positively correlated with LS BMD (*r* = 0.321; *P* = 0.002), LS T-score (*r* = 0.445; *P* < 0.001), FN BMD (*r* = 0.305; *P* = 0.003), and FN T-score (*r* = 0.376; *P* < 0.001). Age was negatively correlated with FN BMD (*r* = −0.213; *P* = 0.042) and FN T-score (*r* = −0.208; *P* = 0.046), although its associations with LS BMD and LS T-score did not reach statistical significance (*r* = 0.097 and *r* = 0.155, respectively; both *P* > 0.05).

**Table 4:** Pearson correlation coefficients between anthropometric variables and BMD parameters in the diabetic group (*n* = 92)

Variable	LS BMD, *r*	*P*	LS T-score, *r*	*P*	FN BMD, *r*	*P*	FN T-score, *r*	*P*
Age (years)	0.097	0.356	0.155	0.140	−0.213	**0.042**	−0.208	**0.046**
BMI (kg/m^2^)	0.321	**0.002**	0.445	**< 0.001**	0.305	**0.003**	0.376	**< 0.001**

Values are Pearson correlation coefficients (*r*) with their *P* values, calculated in the diabetic group. Bold *P* values indicate statistical significance (*P* < 0.05). BMD, bone mineral density; BMI, body mass index; FN, left femoral neck; LS, lumbar spine; *n*, number of participants.

### Bone density distribution by age group in patients with diabetes

Age-stratified analysis within the diabetic group (Table 5) showed that the prevalence of lumbar spine osteoporosis increased with age, from 50.0% in patients younger than 50 years to 56.0% in those aged ≥ 70 years. FN T-scores declined markedly in the oldest age group (≥ 70 years: −1.82 ± 1.13), compared with the 50–59-year group (−0.47 ± 1.03). FN osteopenia was most prevalent in the oldest participants (60.0% at ≥ 70 years).

**Table 5:** Age-stratified bone mineral density parameters in the diabetic group (*n* = 92)

Age group	*n*	LS T-score	LS osteoporosis, *n* (%)	FN T-score	FN osteoporosis, *n* (%)	FN osteopenia, *n* (%)
< 50 years	6	−2.70 ± 2.11	3 (50.0)	−1.70 ± 1.32	2 (33.3)	3 (50.0)
50–59 years	34	−1.74 ± 1.46	9 (26.5)	−0.47 ± 1.03	1 (2.9)	14 (41.2)
60–69 years	27	−2.04 ± 1.28	10 (37.0)	−1.09 ± 0.76	2 (7.4)	15 (55.6)
≥ 70 years	25	−1.96 ± 1.24	14 (56.0)	−1.82 ± 1.13	3 (12.0)	15 (60.0)

T-scores are presented as mean ± SD and categories as *n* (%), with percentages calculated within each age group. Categories follow the WHO T-score criteria. FN, left femoral neck; LS, lumbar spine; *n*, number of participants; SD, standard deviation; WHO, World Health Organization.

## DISCUSSION

This retrospective cross-sectional study examined the association between diabetes mellitus and BMD in 107 adults in Yanbu, Saudi Arabia. The principal finding was that, while the diabetic cohort demonstrated a high absolute burden of reduced BMD—with 39.1% of patients having lumbar spine osteoporosis and 40.2% overall meeting osteoporotic criteria at one or more DXA sites—no statistically significant difference in BMD was identified between the diabetic and non-diabetic groups. BMI was the strongest determinant of BMD in the diabetic cohort, exhibiting a moderate positive correlation with T-scores at both measurement sites, while advancing age was inversely associated with femoral neck bone density.

The absence of a significant difference in BMD between diabetic and non-diabetic participants in the present study aligns with a substantial body of literature on T2DM and bone density. Unlike the robust BMD deficits documented in T1DM, which arise from absolute insulin deficiency and consequent impairment of osteoblastic differentiation and activity [7], the skeletal phenotype of T2DM is characterized more by qualitative bone deterioration than by quantitative bone loss [10]. Several large cross-sectional and longitudinal studies, including the meta-analysis of Qiu *et al*. (*n* = 24,340) [21], have reported BMD values in patients with T2DM that are comparable to or higher than those in non-diabetic controls, yet these patients sustain fractures at disproportionately elevated rates. The 2024 American Diabetes Association Standards of Care accordingly recommend that a T-score of −2.0 in patients with diabetes be interpreted as functionally equivalent to −2.5 in individuals without diabetes, reflecting the inadequacy of standard BMD thresholds to capture the full fracture risk burden in this population [3].

The relatively preserved BMD observed in the diabetic group in the current study may, in part, reflect the protective mechanical effect of higher body weight: the diabetic cohort had a mean BMI of 30.5 ± 7.1 kg/m^2^, consistent with obesity, compared with 29.1 ± 5.9 kg/m^2^ in the non-diabetic group. The moderate positive correlation between BMI and both lumbar spine and femoral neck T-scores in our patients with diabetes (*r* = 0.445 and *r* = 0.376, respectively; both *P* < 0.001) is well supported by Mendelian randomization evidence demonstrating that higher BMI causally increases BMD, potentially mediated through T2DM pathways involving insulin and mechanotransduction [15]. Mechanistically, increased gravitational loading of the skeleton, augmented by adipose-derived estrogen and adipokines, stimulates osteoblast activity and suppresses bone resorption, thereby maintaining or elevating BMD [16]. A Saudi study of women with T2DM similarly reported that increased BMI was significantly protective against both osteopenia and osteoporosis (odds ratio = 0.9 per kg/m^2^; *P* < 0.05) [19].

Despite preserved mean BMD values, the absolute prevalence of osteoporosis in our diabetic cohort was substantial (39.1% at the lumbar spine), which is consistent with findings from the central region of Saudi Arabia—where a BMD study of 327 adults found osteoporosis in 36.8% of patients with diabetes [18]—and with studies from other regional centers reporting overall osteoporosis prevalence rates of 52–64% in DXA-referred populations [5]. The prevalence observed in the current cohort is also broadly consistent with a global meta-analysis reporting an osteoporosis prevalence of 27.67% in T2DM [2]. The higher lumbar spine than femoral neck osteoporosis rate (39.1% vs 8.7%) likely reflects the greater sensitivity of axial DXA for detecting early trabecular bone loss and the known tendency for lumbar spine T-scores to be affected by degenerative changes or osteophytosis, which can artificially inflate lumbar BMD in older adults—potentially leading to underestimation of true osteoporosis prevalence at this site in our cohort [4].

The pathophysiological mechanisms underlying bone loss and fragility in DM are multifactorial. Chronic hyperglycemia drives non-enzymatic glycation of type I collagen, generating pentosidine and other AGE cross-links that alter collagen fiber geometry, impair mineralization, and reduce bone material ductility and toughness [11,12]. AGEs interact with RAGE, triggering NF-κB–mediated inflammatory cascades and MAPK signaling that upregulate pro-resorptive cytokines such as interleukin-6 and tumor necrosis factor-α, while simultaneously promoting osteoblast apoptosis and skewing mesenchymal stem cells toward adipogenic rather than osteoblastic differentiation [13]. Microvascular complications—including cortical bone vascular rarefaction and impaired periosteal blood supply—further contribute to increased cortical porosity and impaired bone repair [12]. These qualitative skeletal deficits are largely invisible to standard DXA-based T-score assessment, which measures only mineral content and not bone matrix quality, explaining the paradox of preserved BMD coexisting with elevated fracture risk in T2DM.

The significant negative correlation between age and femoral neck T-score (*r* = −0.208; *P* = 0.046) and BMD (*r* = −0.213; *P* = 0.042) in our diabetic cohort corroborates established age-related bone loss patterns, whereby the inevitable decline in oestrogen production in postmenopausal women, coupled with diminished osteoblast proliferative capacity and reduced physical activity, accelerates cortical and trabecular bone loss at the femoral neck [22]. The age-stratified analysis in our cohort demonstrated a progressive decline in femoral neck T-scores in patients with diabetes aged ≥ 70 years (mean −1.82 ± 1.13), compared with those aged 50–59 years (mean −0.47 ± 1.03), highlighting older adults with diabetes as a particularly high-risk subgroup for hip fracture, which carries the most severe consequences in terms of mobility loss, institutionalization, and mortality [10,23]. The lack of a statistically significant age–lumbar spine BMD association in our data may reflect the confounding influence of degenerative osteophytic changes at lumbar levels, which are more prevalent in older individuals and can artefactually elevate apparent BMD on DXA imaging [4].

Several limitations of this study warrant consideration. First, the small non-diabetic comparator group (*n* = 15) substantially reduced statistical power to detect intergroup BMD differences, which may have contributed to the non-significant findings in group comparisons. Second, this was a retrospective design relying on clinical records; information on diabetes type, duration, glycaemic control (glycated hemoglobin), antidiabetic medications, vitamin D status, physical activity, and menopausal status—all established determinants of BMD—was not available for analysis. The predominance of women (79.4%) and Saudi nationals (95.3%) in the sample limits generalisability to other demographic groups. Third, the use of T-scores in patients with diabetes may underestimate the true fracture risk, as discussed above; the absence of trabecular bone score (TBS) data, which captures spinal bone microarchitecture beyond BMD, is a notable limitation. Fourth, as a single-center study in an industrial city, the results may not be representative of broader Saudi Arabian or regional populations.

Notwithstanding these limitations, this study provides the first BMD characterization data from adults with diabetes in Yanbu and adds to the growing evidence that reduced bone health is highly prevalent in Saudi patients with diabetes. The clinical implication is that BMD screening should be integrated into the routine management of patients with diabetes, particularly those who are older and of lower body weight. Given that standard T-score thresholds may underestimate fracture risk in diabetes [3], clinicians should consider supplementary assessment tools such as TBS, the Fracture Risk Assessment Tool (FRAX) with a diabetes-specific adjustment, or serum bone turnover markers when evaluating skeletal fragility in this population. Prospective cohort studies with larger, well-characterized samples and longitudinal follow-up are needed to determine incident fracture rates and to elucidate the relative contributions of glycemic control, medication use, and nutritional status to BMD trajectories in Saudi patients with diabetes.

## CONCLUSION

This study demonstrated a high burden of reduced BMD among adults with diabetes mellitus in Yanbu, Saudi Arabia, with 39.1% of patients with diabetes meeting the WHO T-score criterion for lumbar spine osteoporosis and 40.2% having osteoporosis at one or more DXA sites. Although no statistically significant difference in BMD was found between diabetic and non-diabetic groups—likely attributable to the limited size of the comparator group—the data reveal a clinically meaningful degree of skeletal compromise in this population. BMI was the strongest positive predictor of bone density in patients with diabetes, while advancing age was independently associated with femoral neck bone loss. These findings highlight the need for systematic BMD screening, early intervention, and multidisciplinary management strategies targeting bone health in adults with diabetes in this region.

## Data Availability

The anonymized dataset supporting this study is available from the corresponding author upon reasonable request, subject to applicable privacy regulations.

## Personal thanks

The authors would like to thank the Royal Commission Hospital in Yanbu for supporting this research.
